# The Etiological Role of Impaired Neurogenesis in Schizophrenia: Interactions with Inflammatory, Microbiome and Hormonal Signaling

**DOI:** 10.3390/ijms26199814

**Published:** 2025-10-09

**Authors:** Miu Tsz-Wai So, Ata Ullah, Abdul Waris, Fahad A. Alhumaydhi

**Affiliations:** 1 Department of Neuroscience, Jockey Club College of Veterinary Medicine, City University of Hong Kong, Hong Kong SAR 999077, China; 2 Department of Biomedical Science, City University of Hong Kong, Hong Kong SAR 999077, China; 3 Department of Medical Laboratories, College of Applied Medical Sciences, Qassim University, Buraydah 52571, Saudi Arabia

**Keywords:** psychosis, schizophrenia, neurogenesis, inflammation, immune aberrations, androgens, estrogen, HPA axis

## Abstract

Schizophrenia is a prevailing yet severely debilitating psychiatric disorder characterized by a convoluted etiology. Although antipsychotics have been available for over half a century, they primarily mitigate symptoms rather than providing definitive care. This limitation suggests that the neurotransmitter systems targeted by these medications are not the root cause of the disorder. Ongoing research seeks to elucidate the cellular, molecular, and circuitry pathways that contribute to the development of schizophrenia. Unfortunately, its precise pathogenesis remains incompletely understood. Accumulating evidence implicates dysregulated neurogenesis and aberrant neurodevelopmental processes as key contributors to disease progression. Recent advances in proteomics and imaging technology have facilitated the emergence of novel models of schizophrenia, emphasizing the roles of neuroinflammation, sex steroids, and cortisol. This paper aims to organize and map the intercorrelations and potential causal effects between various mechanistic models to gain deeper insight on how these mechanisms contribute to the cause, risks, and symptoms of the disorder. Furthermore, we discuss the potential therapeutic strategies that target these pathological pathways. Elucidating these mechanisms may ultimately advance our understanding of schizophrenia’s etiological foundations and guide the development of curative interventions.

## 1. Introduction

Schizophrenia (SCZ) is a complex psychiatric disorder that affects approximately 0.7% of the population globally. It manifests through positive, negative, and cognitive symptoms that substantially impair daily functioning. The current first-line medication for SCZ is first-generation antipsychotics, which are primarily dopamine D2 receptor antagonists. Despite decades of extensive research, the etiology of SCZ remains elusive, largely due to the heterogeneity of its symptoms and behavioral presentations that obscure its underlying mechanisms.

In recent years, the neurodevelopmental model of SCZ has gained increasing support, proposing that disruptions in neurodevelopmental processes—arising from genetic and environmental factors—contribute to disease onset [1]. Proposed by Bayer et al., the “two-hit hypothesis” of schizophrenia has postulated that genetic mutants and early-life environmental risk factors first heighten the brain’s vulnerability to the disorder, followed by subsequent environmental insults that precipitate immune and neurochemical dysregulation, ultimately triggering SCZ symptom emergence by early adulthood [2,3].

DSM-V diagnostic criteria for SCZ range from delusions, hallucinations, disorganized speech, and/or catatonic behaviors [4]. Besides behavioral abnormalities, multiple structural alterations have been identified in SCZ patients. Various studies have demonstrated brain network disconnection [5] and reductions in gray matter volume, especially in the right dorsolateral prefrontal cortex (DLPFC) [6]. These structural abnormalities have been correlated with cognitive deficits characteristic of SCZ [7].

Among the earliest neurobiological findings in SCZ is neurotransmitter imbalance. It has been reported that several neurotransmitters and pathways have been implicated in the SCZ pathophysiology. The dopaminergic (DA) system is the most extensively studied neurotransmitter involved in SCZ pathophysiology. The dopamine hypothesis of SCZ, proposed a few decades ago, posits that SCZ arises from hypoactivity in the mesocortical pathway and hyperactivity in the mesolimbic pathway [8]. Hallucination, one of the most prevalent positive symptoms in SCZ, has been proposed to be mediated by striatal DA hyperactivity, as demonstrated by both animal models and human imaging studies [9,10]. Contrary to prior research, more recent work by Yun et al. has revealed that D1 receptor (D1R)-expressing striatal projection neurons, instead of D2 receptor populations, are more strongly associated with psychosis-like behaviors [11].

Another key neurotransmitter involved in SCZ pathophysiology is γ-aminobutyric acid (GABA), the key inhibitory neurotransmitter in the central nervous system (CNS). The disruptions in excitatory/inhibitory (E/I) balance have been proposed to be the core pathology in SCZ [12]. Lower GABA levels have been associated with both disease severity and cognitive impairments in SCZ. The E/I imbalance is also contributed to by glutamate hyperfunction in multiple brain circuits, particularly through N-methyl-D-aspartate glutamatergic receptor (NMDAR) dysfunction. NMDAR hypofunction impairs parvalbumin (PV-positive) inhibitory interneurons, highlighting the importance of inhibitory actions in maintaining normal brain functions [13]. NMDAR agonists have shown efficacy in ameliorating negative symptoms, whereas NMDAR antagonists can induce schizophrenia-like manifestations [12,14].

This review consolidates and examines the potential roles of impaired neurogenesis and neuroinflammation in SCZ pathogenesis, alongside disruptions in gut microbiota composition, HPA axis function, and sex hormone regulation. Collectively, these interconnected systems may converge to drive the onset and progression of schizophrenia.

## 2. Neurogenesis and Neurodevelopment

Neurogenesis, is key process in neurodevelopment, refers to the generation of newborn neurons from neural stem cells (NSCs) or neuronal progenitor cells (NPCs) [15]. Aberrations in adult neurogenesis can compromise mental well-being and serve as a predisposition to psychiatric disorders [16]. As a neurodevelopmental disorder, SCZ has been associated with impaired postnatal neurogenesis in both animal models exhibiting SCZ-like behaviors [17] and human patients [18,19], underscoring their probable linkage.

Neurogenesis is a highly intricate process regulated by different cellular signals, neurotransmitters, and transcription factors. Alternations in neurogenesis and related genes have been reviewed by Iannitelli et al., who demonstrate such aberrations correlate with an increased risk of schizophrenia [20]. Their study highlights key SCZ susceptibility genes, such as Neuroregulin (NRG1), Reelin, and *DISC1*, that modulate neurogenic signaling pathways.

Recent advances, including in vitro cell culturing and bioinformatic approaches, have provided deeper insight into neurogenesis-SCZ associations. Neurospheres derived from SCZ subjects have exhibited a markedly reduced self-renewal capacity among all generations. They are also significantly smaller from passage 2 onwards with a progressive reduction in size throughout passages [21]. Pathway analysis of the neurons obtained from the olfactory epithelium of patients has revealed downregulation of signaling pathways in cell proliferation, growth, and synaptic transmission modulation, especially featuring SCG2, which is similarly downregulated in the DLPFC of postmortem patient samples [22]. Furthermore, cerebral cortex organoids generated from individuals with 22q11.2 deletion syndrome shows delayed NPC differentiation, a bias towards progenitor states, and reduced neurite complexity compared to controls. Pathway enrichment analysis reveals patient’s iPSC-derived neurons have a downregulation in neuronal maturation and upregulation in negative controls of neurogenesis [22]. Consistently, human-induced pluripotent stem cells (hiPSCs) derived from 15q11.2 deletion-bearing patients, a risk factor for SCZ, was differentiated into cortical NPCs, then differentiated and co-cultured with rat astrocytes, as described by Habela et al. [23]. Several distinctions have been observed in 15q11.2 deletion-derived samples, including alternations in cell type fate, reduced neurite complexity, and notably, delayed maturation in GABAergic neurons [23]. These demonstrate the robust correlations between disturbed neurodevelopmental processes and SCZ at a cellular level.

Emerging evidence also implicates other pathways such as neuroinflammation, oxidative stress, and neuroendocrine signaling in SCZ pathology. These processes, shaped by genetic predispositions and environmental influences, can disrupt neurodevelopmental trajectories and neurochemical homeostasis, leading to structural brain alterations. Despite accumulating evidence, in the status quo, few studies have comprehensively integrated these mechanistic domains. Therefore, this paper aims to elucidate the interplay among neurogenesis, inflammation, microbiome dysregulation, HPA axis activity, and hormonal signaling, providing a multidimensional perspective on SCZ etiology. A holistic synthesis of these interrelated mechanisms may advance our understanding of the disorder’s pathogenesis and guide the development of more targeted therapeutic interventions.

## 3. Immune Dysregulation in Schizophrenia

The earliest conceptualization of immune dysregulation in schizophrenia was introduced by Smith and Maes through the monocyte-T lymphocyte theory of SCZ in 1995 [24]. They postulated that monocytes and T cells are activated in the acute phase of SCZ, as reflected by the heightened levels of activation markers. This acute inflammation can cause NMDAR malfunctioning and therefore neurotoxicity and chronic neuroinflammation—potentially serving as a key pathogenic mechanism [25,26].

Subsequent meta-analyses have consistently reported elevated cytokines and pro-inflammatory markers in SCZ patients [27], particularly involving the NF-κB (necrosis factor kappa B) pathway. NF-κB acts as a master regulator of inflammation, mediating the release of pro-inflammatory cytokines, chemokines, and reactive nitrogen species (RNS), therefore establishing a self-perpetuating positive feedback loop [28]. Recently, a correlation study has demonstrated significant correlations between interleukin-6 (IL-6; r = 0.39) and Oncostatin M (OSM; r = 0.51), with OSM known to facilitate IL-6 production [29]. In drug-naïve FEP patients, elevated peripheral IL-6 and IL-2 levels have also been observed [30]. Increased IL-6 and soluble IL-2 receptor levels in unaffected first-degree relatives of SCZ patients suggest a genetic predisposition to inflammatory vulnerability [31,32].

Apart from peripheral markers of inflammation, differential gene expression (DEG) analysis of human schizophrenic postmortem samples has revealed immune-related transcriptional signatures [33]. Weng et al. have screened and identified 151 SCZ-immune-related genes and, through machine learning approaches, distilled 17 signature genes capable of distinguishing SCZ from control samples with an average ROC AUC of ≥0.85 [33]. These findings underscore the profound involvement of immune dysregulation in SCZ pathology.

Environmental risk factors—prenatal, perinatal, and postnatal—may further potentiate neuroinflammation. Childhood distress, including maltreatment and physical abuse, has been shown to activate the immune system and is closely associated with schizophrenia vulnerability. Corsi-Zuelli et al. demonstrated that the increased level of TGF-β is associated with exposure to early-life stress, especially in patients, acting as a neuroprotective response [34]. Aside from stress, peripheral immune activation resulting from infection can induce neuroinflammation via blood–brain barrier permeability, particularly when pre-existing vulnerability is present due to genetic or oxidative stress-related factors.

These environmental factors can induce epigenetic modifications, bringing up a gene-environmental interaction and increasing the odds of SCZ through differential gene expression [34]. DNA methylation, catalyzed by DNA methyltransferases (DNMTs), represents a key mechanism. Alternations in the DNA methylation profile in FEP subjects have been observed in a meta-analysis, with an overall hypomethylation and a relative hypoexpression of *DISC1* [35]. *DISC1* (Disrupted-in-Schizophrenia 1) gene is one of the most studied SCZ-susceptible genes, which has been found to be associated with aberrant neurogenesis, including reduced proliferation, over-branching, and structural changes, leading to premature frontotemporal cortex maturation [15]. *DISC1* modulates the Wnt/β-catenin signaling pathway, which is crucial for neuronal proliferation [36]. Impairment of this pathway exacerbates cytokine- and complement-mediated neurotoxicity, increasing vulnerability to inflammation-driven neuronal injury [37]. These findings highlight the bidirectional interactions between neurodevelopmental and immune signaling pathways.

At the molecular level, neuroinflammation can impair multiple cellular pathways vital for neuronal functioning. Pro-inflammatory cytokines activate NF-κB, promoting excessive production of reactive oxygen species (ROS) and nitrogen species (RNS). Cells have an innate ability to regulate redox balance through antioxidants. Under normal physiological conditions, low ROS levels are essential for cellular purposes like signal transduction. However, excessive ROS and RNS overwhelm the cell’s antioxidant defenses, leading to oxidative stress (OS). Indeed, meta-analytic data reveal reduced total antioxidant status in FEP patients (*p* < 0.01), reversible following antipsychotic treatment across 44 studies [38]. Prolonged OS can induce lipid peroxidation, protein oxidation, and dopamine autoxidation, further amplifying ROS generation. Furthermore, OS contributes to hypomyelination, which has been observed in postmortem SCZ brains [39].

ROS accumulation is intimately associated with mitochondrial dysfunction (MD), as ROS can damage key regulators of ATP production such as NADH dehydrogenase and cytochrome c oxidase (COX), resulting in mitochondrial DNA (mtDNA) damage and disrupted energy metabolism [40]. Participation of MD in SCZ pathology has been widely discussed, supported by transcriptomic and meta-analytic findings from postmortem brain tissues [41,42]. Given mitochondria’s central role in neuronal energy metabolism, their dysfunction can compromise synaptogenesis and synaptic transmission—processes essential for neurodevelopment [41]. Notably, *DISC1*, which we have previously mentioned, is localized to mitochondria-associated endoplasmic reticulum (MAMs), where its dysregulation can promote abnormal calcium accumulation within mitochondria, further exacerbating cellular stress [41].

## 4. Gut Microbiome and Schizophrenia

Recent studies have revealed the bidirectional relationship between mitochondria and gut microbiome in the pathophysiology of schizophrenia [43]. The gut–brain axis (GBA) refers to the intricate bidirectional communications between gut microbiota and the CNS [44]. Disturbances of the axis have been observed in different neurodevelopmental disorders [45] and mood disorders [46], including SCZ. The GBA operates through neuronal, immune, and endocrine pathways, thereby integrating environmental influences with neuroinflammatory processes that contribute to disease pathology [45].

Meta-analytic findings in 2022 have revealed alterations in the gut microbiota composition of individuals with schizophrenia [47]. Specifically, a relative increase in lactic acid-producing bacteria, such as *Escherichia*/*Shigella*, has been observed in SCZ [47]. This leads to a drop in gut and brain pH value, fostering neurotoxicity and mitochondrial dysfunction [48]. The imbalance of the microbiome profile in the gut, also referred to as dysbiosis, can lead to increased gut permeability, enabling translocation of microbial products and inflammatory mediators, and consequently, neuropsychiatric disorders [49]. Altered microbiota composition is also correlated with reduced gray matter volume and regional homogeneity in SCZ patients, indicating structural and functional correlates of microbiome disruption [50].

Short-chained fatty acids (SCFAs) are the products from microbiomes that could cross the BBB and influence CNS activity directly. Dysbiosis alters the levels of produced SCFAs, producing wide-ranging effects [51]. Butyric acid, primarily synthesized by *Coprococcus*, is notably reduced in SCZ [47]. Butyrate is crucial for maintaining gut barrier integrity, thereby preventing microbial translocation and systemic inflammation [45,47]. It also mediates the hypothalamic–pituitary–adrenal (HPA) axis. Administration of butyrate to stressed mice helps alleviate their stressful behaviors, potentially through histone acetylation at the *CRHR2* gene promoter [52,53]. However, another longitudinal study failed to show the decrease in butyric acid levels during the prodromal phase of SCZ but found lower levels of valeric acid and caproic acid instead [54]. Higher levels of valeric acid in the brain have been shown to decline following exercise, a neuroprotective mechanism against neuroinflammation [55]. Interestingly, valeric acid administration has been found to protect dopaminergic neurons in Parkinson’s disease models by preventing rotenone-induced apoptosis, glial activation, and OS [56]. These mixed findings highlight the need for further investigation into SCFA roles in neuroprotection.

Disrupted GBA can be detrimental to neuronal survival and neurogenesis. Induced dysbiosis following traumatic brain injury hinders immediate reparative neurogenesis by inhibiting monocyte and lymphocyte infiltration, resulting in enhanced neurodegeneration [57]. Another study has found that mice supplemented with probiotics have higher levels of neurogenic Ly6C(hi) monocytes in the CNS when compared to antibiotic-treated ones, which are positively associated with neurogenesis [58].

Probiotics are microorganisms believed to ameliorate microbiota dysbiosis upon administration [59]. They exert several beneficial effects on GBA, such as reducing systemic inflammation and gut permeability and increasing the production of SCFAs [60]. However, the efficacy of probiotics as adjunctive therapy remains inconclusive, partly due to small sample sizes in clinical trials and systematic reviews (Table 1).

## 5. Cortisol and the HPA Axis

The hypothalamic–pituitary–adrenal (HPA) axis constitutes a central stress-response system modulated by immune and endocrine signals. Upon activation, the paraventricular nucleus of the hypothalamus would secrete corticotropin-releasing hormone (CRH), which stimulates adrenocorticotropic hormone (ACTH) release from the pituitary gland. ACTH subsequently induces cortisol secretion from the adrenal cortex [63]. Under normal conditions, cortisol exerts negative feedback on CRH and ACTH secretion, maintaining homeostasis. In addition to its circadian regulation, cortisol release increases transiently in response to stressors.

Physiologically, glucocorticoids exert immunosuppressive effects. Pro-inflammatory cytokines can upregulate HPA axis activity, resulting in elevated cortisol levels [64]. Chronic stress and prenatal exposure to glucocorticoids may lead to a dysregulation of the immune response via glucocorticoid receptor desensitization, impaired HPA feedback, and sustained pro-inflammatory cytokine release, while concurrently suppressing anti-inflammatory signaling [65]. This reciprocal interaction between stress and inflammation underscores the HPA axis’s contribution to schizophrenia pathogenesis.

The HPA axis is also intricately linked with neurotransmitter systems, particularly dopaminergic signaling. Cortisol and dopamine (DA) levels rise concurrently following stress exposure [66]. Cortisol modulates DA synthesis by enhancing *tyrosine hydroxylase* (TH) expression, the rate-limiting enzyme in DA production, and potentiating dopaminergic activity in subcortical regions [67]. Prenatal stress-induced neonatal hippocampal lesions can cause increased DA agonist response after pubertal maturation, resulting in SCZ-like phenotypes [68]. Conversely, dopaminergic agonists such as apomorphine can activate the HPA axis, elevating ACTH and cortisol in patients with schizophrenia and major depressive disorder [69].

Stress and the HPA axis have long been known to suppress neurogenesis. High doses of glucocorticoids are proposed to exert their actions on glucocorticoid receptors on neuronal progenitor cells, which can disrupt their proliferation and differentiation properties with a long-term effect [70]. The hippocampus, the prominent site for adult neurogenesis, is exceptionally sensitive to stress due to its high concentration of glucocorticoid receptors and hence is particularly vulnerable to stress-associated damage [71]. Glucocorticoids can impact various cellular pathways that have been implicated in SCZ and neurogenesis. Firstly, glucocorticoids can inhibit proliferation and differentiation by increasing Dickkopf1 (DKK1) expression, an antagonist of the Wnt signaling pathway essential for NSC proliferation and differentiation [72]. *DISC1*, another schizophrenia susceptibility gene, modulates canonical and non-canonical Wnt signaling via GSK-3β regulation [36]. Dysregulation of these pathways under glucocorticoid influence suggests a mechanistic link between stress, impaired neurogenesis, and schizophrenia.

Activity repression in NSCs has been observed after the administration of cortisol. Glucocorticoids also inhibit proliferation specifically by upregulating p21 and p16, cell cycle blockers, and suppressing differentiation by reducing the expression of TrkB receptors, which are receptors of highest affinity with brain-derived neurotrophic factor (BDNF) [73]. The downregulation of neurogenesis, induced by dexamethasone, simultaneously downregulates four enzymatic antioxidants in vitro, while exogenous administration of antioxidants can restore reduced TrkB expression to normal [74]. Another study shows that exposure to dexamethasone only reduced NSCs’ proliferation but had no effect on other aspects of neurogenesis. Yet, the epigenetic changes correlated with glucocorticoid and senescence are retained in daughter NSCs, which have no prior exposures to OS. These daughter cells not only have an increased vulnerability towards OS but also are more likely to undergo apoptosis after exposure to ROS-associated stimuli [75]. These have demonstrated the robust relationship between OS, early-life HPA axis activation, and neurogenesis. Intriguingly, inhibition of neurogenesis by Valganciclovir, an anti-proliferating agent, was found to multiply HPA-axis response under acute stress [76]. Chronic stress, on the other hand, produces habituation and blunted cortisol responsiveness, consistent with HPA feedback impairment observed in schizophrenia [77]. Tamoxifen-induced neurogenesis has been shown to normalize HPA activity following chronic stress [78], while neurogenic stimulation restores basal cortisol levels in chronic stress models [78,79]. Collectively, these findings demonstrate a reciprocal regulatory relationship between neurogenesis and HPA axis function.

Altered HPA activity is a well-established feature of schizophrenia. Schizophrenic patients have significantly higher serum baseline cortisol levels than Major Depressive Disorder (MDD) patients and controls; higher cortisol levels are positively correlated with higher Brief Psychiatric Rating Scale (BPRS) scores, showing that elevated cortisol levels reflect higher symptom severity (R = 0.36, *p* < 0.01) [80]. As higher cortisol shows a higher state of stress, it could be concluded that the high stress state in schizophrenia interacts with higher cortisol levels, which increases the severity of the disorder. Cortisol baseline levels are mostly correlated with an array of negative symptoms, positive symptoms, and disorganization, and this mixed result could be explained by the complex interaction between the HPA axis and the brain [81]. A comprehensive meta-analysis has shown that antipsychotic treatment is effective in reducing the cortisol levels and HPA axis activity in SCZ patients, potentially due to the interactions between DA receptors, inflammatory response, and glucocorticoid receptor-mediated gene transcriptions [82].

The cortisol awakening response (CAR)—a rapid rise in cortisol secretion within 30–60 min after waking—is attenuated in schizophrenia [83,84]. Systematic reviews report blunted CAR in both schizophrenia (g = 0.556) and FEP patients (g = 0.544), supporting the hypothesis of chronic HPA dysregulation associated with prolonged stress exposure [85].

## 6. Sex Steroids and Sexual Dimorphism

Schizophrenia exhibits clear sexual dimorphism in onset, symptomatology, and severity. SCZ onset ages are typically earlier for men (peaks at age 18–24) (ages 18–24), whereas females display a secondary peak around menopause (ages 45–50) [86]. Men are also 1.4 times more likely to be affected by SCZ than women and often exhibit more pronounced structural abnormalities, such as enlarged ventricles [87,88]. These observations shed light on the roles of sex steroids in SCZ, particularly estrogen and androgen.

It has been proposed that the production of estrogen by ovaries in women has raised the threshold of women from having SCZ [89]. Lower estradiol levels in serum have been noted in female SCZ patients, along with dampened fluctuations during the menstrual cycle [90]. Positive symptom severity was found to correlate inversely with estrogen levels [91]. Furthermore, a literature review has revealed that hypoestrogenism due to oophorectomy can lead to cognitive decline, such as verbal memory, poorer performance in the Wechsler Memory Scale, and higher risks of dementia later in life, showing that estrogen could modulate cognitive symptoms in SCZ [92]. This leads to the proposal of the estrogen hypothesis, in which hypoestrogenism is correlated with higher risks of SCZ. Adjunctive estradiol treatment to antipsychotics has been found to reduce the severity of negative symptoms and require a lower dosage [93]. A clinical trial has found improvements in cognitive capacity after 6 weeks of Raloxifene (selective estrogen receptor modulator, SERM), used adjunctively with L-type Ca^2+^ channel blocker [94]. Meta-analytic data corroborate the efficacy of Raloxifene-adjunctive therapy in mitigating most SCZ symptom domains and severity. SERMs have been found to not only modulate estrogen receptor pathways, activating downstream signaling such as the PI3K/Akt pathway, but also exert protective effects against neurodegeneration through lowering ROS levels and guarding oxygen-glucose-derived astrocytes [95,96].

Estrogen has long been proposed as a neuroprotective factor. The actions of estrogen and its derivative estradiol on neurogenesis include increasing mature neuron counts, promoting synaptic density, and enhancing neuronal growth by acting with growth factors and growth factor receptors [97]. When endogenous estrogen binds to G-protein coupled estrogen receptor (GPER), it activates the epidermal growth factor receptor (EGFR) and promotes downstream signaling cascades including PKA activation, Ras/PI3K, and ERK/Akt that phosphorylate receptors and promote expression of cell survival and neurogenesis-associated genes [98]. Upon the activation of GPERs in the dorsal hippocampus, which are abundantly expressed in hippocampal neurons, memory consolidation is facilitated via the JNK signaling pathway and spinogenesis [99]. Deficiency of GPER has been noted in adult mice to decrease neuronal proliferation in the hippocampus via ERK/PKA/IGF-1 pathways and consequently causes cognitive decline, exhibiting the importance of estrogen in modulating neurogenesis [100].

Estrogen regulates the neurotransmitter signaling systems in the brain, including dopamine (DA), serotonin, and glutamate. It has been reported that estradiol has a female-specific effect on enhancing striatal DA, though the mechanism remains opaque. Acute exogenous estradiol has been found to increase the turnover rate of DA and downregulate D2 receptor binding [101,102]. Another study has reported 17β-estradiol mediates dopaminergic neuron activities in the ventral tegmental area (VTA) by enhancing D2/D3 receptor sensitivity [103]. Given dopamine’s central role in schizophrenia pathophysiology, these findings highlight a critical intersection between estrogen and dopaminergic signaling.

Androgen is a group of steroid hormones with a substantially higher amount in males. Examples include testosterone, dehydroepiandrosterone (DHEA), and DHEA-sulfate (DHEA-S). They bind to androgen receptors (AR) that are present in hippocampal neurons and glia. Meta-analysis has revealed SCZ patients have elevated levels of free testosterone and DHEA-S, suggesting that higher testosterone levels may take part in SCZ pathology [104]. Higher testosterone levels are also correlated with increased excitement, hostility, and impulsive behaviors, while inversely correlated with negative symptoms in non-aggressive male SCZ patients [105]. However, these associations appear sex-dependent, as similar correlations were not observed in female patients [106].

The influence of androgens on hippocampal neurogenesis remains contentious. Genetic knockout of AR can lead to hypoactivation in the glutamatergic system, an indispensable part of LTP induction [107]. Yet, conversely, orchidectomy has been shown to enhance synaptic transmission and LTP in the mossy fiber pathway [108]. Given testosterone’s ability to aromatize into estradiol, future studies must control for this conversion to delineate testosterone’s independent effects.

Sex hormones are of prominent importance in the regulation of the HPA axis. While testosterone inhibits the HPA axis, estradiol has an ambiguous role in it [109]. A recent study has found positive correlations between the cortisol/cortisone profile in SCZ patients and symptom severity [110]. Nevertheless, meta-analyses reveal no significant sex-based differences in baseline cortisol levels among SCZ patients, likely reflecting limited sample power [82]. Early-life trauma and chronic stress have been proposed to have a sex-dependent effect on SCZ symptom presentation, severity, and onset age, owing to the interactions between the HPA axis and estrogen [111,112,113].

## 7. Hypothesis: The Neurogenesis-Inflammatory-Gut Dysbiosis Framework of Schizophrenia

**Figure 1:**
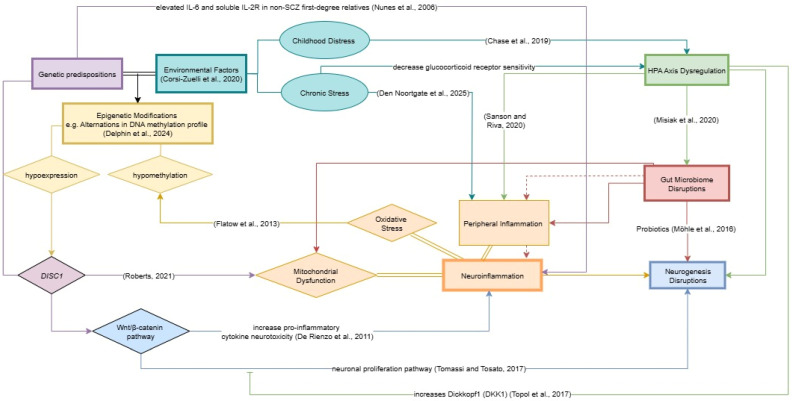
Neurogenesis-inflammatory-dysbiosis hypothesis of schizophrenia pathogenesis. In addition to the established view that schizophrenia pathology stems from aberrant neurogenesis [17,18,19,20,21,22,23] and its interactions with neuroinflammation [24,25,26,27,28,29,30,31,32], emerging evidence suggests additional modulatory factors. Environmental exposures across the lifespan—from childhood adversity to chronic stress—are linked to dysregulation of the HPA axis [80,81,82,83,84,85] and heightened neuroinflammatory activity [34,114,115,116]. Peripheral inflammation, triggered by chronic stress [116], HPA dysfunction [65], or microbial imbalance [45,47,61], can amplify central neuroinflammation in schizophrenia [117,118]. HPA axis abnormalities, which interact closely with neurogenesis impairment [70,71,72,73,76,77,78,79], may also disrupt gut microbiota composition—another well-established risk factor for schizophrenia [119]. Gut microbiota alterations, as reported in multiple studies [45,47,50,60,61,62], facilitate peripheral–CNS inflammatory crosstalk (indicated by red arrows). Increased intestinal permeability permits the translocation of cytokines and chemokines into systemic circulation, subsequently crossing the blood–brain barrier and initiating neuroinflammatory cascades [120]. Dysbiosis is also directly linked to reduced neurogenesis [58]. Furthermore, gene–environment interactions—mediated through epigenetic modifications such as DNA hypomethylation and *DISC1* downregulation [15]—may connect genetic susceptibility with neurodevelopmental impairment. *DISC1* dysregulation affects Wnt/β-catenin signaling [36,37], mitochondrial integrity [41,42], and cytokine-induced neurotoxicity [37], further propagating neural dysfunction. Collectively, these interconnected mechanisms form a multidimensional framework through which genetic, immune, neuroendocrine, and microbial perturbations synergistically contribute to schizophrenia pathogenesis. Abbreviations: HPA: Hypothalamic–Pituitary–Adrenal; IL: Interleukin; NSCs: Neural Stem Cells; SCZ: Schizophrenia.

Various attempts have been made to construct integrative etiological models of schizophrenia, incorporating the complex interactions among neurodevelopmental, immune, and endocrine systems. likely originates from cumulative disturbances throughout the lifespan—beginning with early-life stressors, neurodevelopmental disruptions, and aberrant hypothalamic–pituitary–adrenal (HPA) axis regulation, compounded by chronic neuroinflammation, gut–brain axis (GBA) dysfunction, and sex hormone imbalances. Here, we propose a conceptual model—the Neurogenesis–Inflammatory–Gut Dysbiosis Framework—that delineates how these mechanisms may converge to drive disease onset and progression (Figure 1).

**Figure 2:**
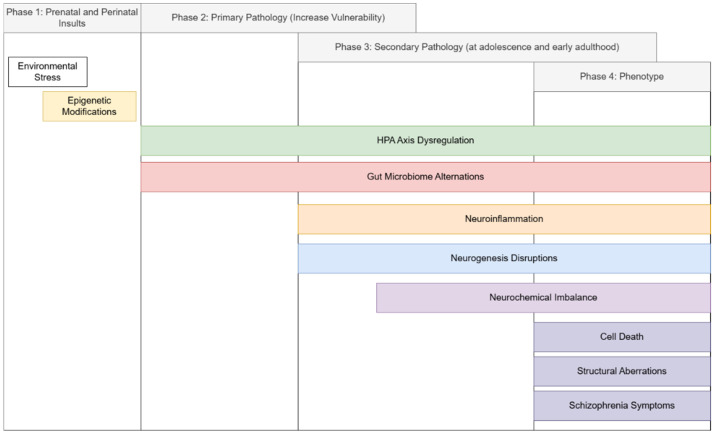
Phases of schizophrenia. The disease course of schizophrenia is proposed to be divided into four phases, with reference to Bayer et al.’s [2] double-hit model of schizophrenia. Phases one and two are the predisposition phases, when individuals are building up vulnerability towards schizophrenia. Phase three takes place at adolescence and early adulthood, when the secondary neurodevelopment stage hits, and sex hormone fluctuations at this stage can contribute to susceptibility. The accumulation of “vulnerability components” leads to the outbreak of schizophrenic symptoms and pathology in phase 4.

Schizophrenia pathophysiology should be conceptualized as a phasic and recursive process that unfolds in developmental stages, as illustrated in Figure 2.

## 8. Future Interventions

Despite extensive research, first-line schizophrenia treatments remain largely confined to dopamine D2 receptor (D2R) antagonists—typical antipsychotics—which primarily alleviate positive symptoms such as hallucinations and delusions but do not address underlying pathology. Moreover, their non-specific pharmacodynamics and propensity to induce oxidative stress contribute to extrapyramidal side effects (EPS) [121]. Consequently, recent therapeutic efforts have sought to target upstream mechanisms such as neuroinflammation, oxidative stress, and impaired neurogenesis to enhance efficacy and reduce adverse effects (Table 2).

### 8.1. Atypical Antipsychotics

Atypical antipsychotics (APP) are primarily D2R and 5-HT receptor 2a inhibitors; hence, they are less likely to induce EPS and possess broader pharmacological effects [128]. A study with 48 SCZ patients reported significant reductions in serum malondialdehyde (a lipid peroxidation marker) and ascorbic acid after eight weeks of treatment, correlating inversely with symptom severity [122]. Similarly, in vitro experiments revealed that risperidone suppresses nitric oxide and pro-inflammatory cytokines (IL-1β, IL-6, TNF-α) in IFN-γ–activated microglia, whereas haloperidol did not, suggesting anti-inflammatory microglial modulation [123]. Meta-analyses confirm risperidone’s capacity to attenuate chronic neuroinflammation, though effects are less pronounced in first-episode psychosis [136].

Concerning the neuroprotective properties of APP, studies have shown that APP might be favorable to neuronal survival, neurogenesis, and dendritic development. Ono et al. have shown that the administration of Olanzapine can significantly restrain neuronal apoptosis after MK-801 exposure or nutrient deprivation, which secures neuronal survival [124]. Clozapine increases *BDNF* expression in ketamine-induced SCZ models and promotes dendritic branching in doublecortin-positive neurons of the dentate gyrus [125]. An increasing trend of nestin concentration in the hippocampus, a marker for newborn neurons, is also observed post-Clozapine treatment [126]. Administration of Risperidone might support neuronal survival by preventing further frontal white matter loss in SCZ subjects [127]. A study with 75 patients also discovered that APPs like Clozapine and Olanzapine can exhibit higher neuroprotective properties than typical antipsychotics by a higher increase of 17β-estradiol in serum posttreatment [137].

### 8.2. Exogenous Stem Cells

The neuroprotective role of exogenous neural stem cells (NSCs) has been suggested by Ono et al. [124]. After exposure to MK-801 or serum/nutrient deprivation, approximately half of the untreated cortical neurons experienced apoptosis. However, for samples with exogenous NSCs, 60–70% of cells survived. The study has proposed that exogenous NSCs increase the net intensity of phosphorylated Akt, which was originally lowered after neuronal damage. This is confirmed by the addition of the Akt pathway inhibitor LY294002. This shows that exogenous NSCs could be a way to increase neuronal survival post-injury [124]. Attempts at transplanting exogenous NSCs to mice or rat models of cerebral ischemia have shown positive results in increasing neurogenesis [129,130,131,132,134] and proliferation [129] and reducing symptomatic inflammation [133]. This shows that exogenous NSCs may not only be beneficial to neurogenesis but also to treating neuroinflammation. In a study of NSCs transplantation to tauopathy model mice, improvements in spatial working memory have been observed, eliciting the possible merits of NSCs for cognitive improvements [135].

Mesenchymal stem cell (MSC) transplantation has also shown promise. In a ketamine-induced SCZ mouse model, intracerebroventricular MSC administration improved social and cognitive performance, increased hippocampal doublecortin-positive cell counts, and upregulated neuroprotective pathways involving β-estradiol, PDGF-β, and TGF-β1. MSC treatment concurrently reduced IL-1β and increased IL-17 expression [125]. These findings highlight the potential of stem cell–based therapies to target both neurogenesis and inflammation.

However, translation to clinical applications necessitates caution. Stem cell transplantation carries risks, including immune activation, tumorigenicity, and ectopic proliferation [155,156,157,158]. Comprehensive preclinical safety assessment and large-scale clinical trials are essential before therapeutic implementation. Besides physical concerns, the use of stem cells in treatment and research remains controversial and would need to be further investigated in later studies.

### 8.3. Anti-Inflammatory Agents

Given the centrality of neuroinflammation in schizophrenia, anti-inflammatory adjunctive treatments have attracted growing attention. A meta-analysis has revealed that anti-inflammatory agents, when used as adjunctive therapy, can improve the effectiveness of antidepressant treatment in patients with depression or depressive symptoms, prompting similar trials in psychosis [159]. These anti-inflammatory agents range from non-steroidal anti-inflammatory drugs (NSAIDs), statins, cytokine inhibitors, and glucocorticoids, which are of different mechanisms.

Minocycline, for instance, is one of the most researched novel treatments for SCZ. It is an NSAID that can penetrate the blood–brain barrier (BBB) with high potency of inhibiting reactive microglia [160,161]. Multiple randomized controlled trials (RCTs) have examined its efficacy as an adjunct to first- or second-generation antipsychotics (summarized in Table 3). Most studies report significant improvement in negative symptoms and general psychopathology [152,153,154,162,163,164], with only one trial reporting null effects [165]. Minocycline’s benefits may derive from suppression of nitric oxide and cytokine signaling [165,166], modulation of microglial phenotype toward M2 polarization, and upregulation of the TrkB/BDNF pathway [138,139].

In addition to anti-inflammatory medications, vitamin D, with anti-inflammatory properties, has also shown therapeutic promise. Two double-blinded RCTs demonstrated improved cognitive performance following supplementation [167,168], potentially mediated through regulation of *NRG1* signaling [142]. Co-administration of vitamin D with probiotics further reduced C-reactive protein (CRP) levels and improved cognitive function [141,142]. Another trial found decreased lipid peroxidation and enhanced total antioxidant status, alongside improvements in general schizophrenia symptomatology [140]. It is postulated that vitamin D can reduce oxidative stress markers and hence lead to a neuroprotective effect, which has been vastly investigated in AD/HD [169]. Larger-scale studies are warranted to clarify the neurochemical mechanisms and clinical benefits of vitamin D in psychosis.

**Table 3:** (**a**) Efficacy of Minocycline in SCZ patient clinical trials. (**b**) Efficacy of Minocycline in SCZ patient clinical trials. (*n* = 10).

(a)					
Study	Study Methods	Durations	Subjects	Outcome	Interpretation
[162]	DB, RCT, Adjunct MC (200 mg/day)	8 weeks	SCZ patients (*n* = 94)	Consistent improvement in negative symptoms in the MC group, significantly higher than that of the placebo group.	Negative symptoms
[152]	DB, RCT. Adjunct MC (200 mg/day)	12 months	SCZ patients (*n* = 24)	Significant improvement in CGI scores of the MC group only. Significant reduction in the PANSS total score and positive, negative, and general psychopathology subscales in the MC group only. Reduced tracer intake in fronto-temporal areas when compared with control.	MC may prevent brain alterations observed in early stages of disorder. Protective against gray matter reduction.
[153]	DB, RCT. Adjunct MC with RIS (200 mg/day)	8 weeks	SCZ patients (*n* = 35)	From baseline to week 4, no significant differences. At week 8, a significant reduction in negative symptom scores was observed.	MC can be used to treat negative symptoms.
[154]	DB, RCT. Adjunct MC with RIS (200 mg/day)	8 weeks	SCZ patients (*n* = 38)	Time x treatment interaction for negative, general psychopathology, positive subscales, and total PANSS scores is significant. The MC group predicts negative and positive symptoms significantly.	MC is a tolerable short-term add-on for RIS.
[163]	DB, RCT. Adjunct MC (200 mg/day)	6 months	SCZ patients (*n* = 54)	Minocycline was well tolerated, with few adverse events. It showed a beneficial effect on negative symptoms and general outcome (evident in SANS and CGI). A similar pattern was found for cognitive functioning, mainly in executive functions (working memory, cognitive shifting, and cognitive planning).	Overall, the findings support the beneficial effect of MC add-on therapy in early-phase schizophrenia.
[164]	DB, RCT. Adjunct MC (200 mg/day) with RIS	16 weeks	SCZ patients (*n* = 63)	Significant improvements on total scores, negative subscale scores, and attention domain compared to the placebo group. Better treatment response than placebo.	Considerable negative symptom adjunct treatment
[165]	DB, RCT. Adjunct MC (200 mg/day for 2 weeks, then 300 mg/day for 12 months)	12 months	SCZ patients (*n* = 207)	No effects on any symptom scores, gray matter volume, IL-6, or CRP levels.	MC has shown no effects on SCZ treatment.
[166]	DB, RCT. Adjunct MC (100 mg and 200 mg/day) with RIS	3 months	SCZ patients (*n* = 57)	Significant improvement of cognitive domains (information processing speed, vigilance, working memory, verbal learning and memory, reasoning and problem solving); significant decrease in IL-1β, IL-6, and TNF-α in the high-dosage group compared with the two other groups, but not in the low-dosage group compared with the control. A decrease in IL-1β and IL-6 in the high-dosage group correlated with improvements in cognitive symptoms.	Cognitive deficits ameliorated after adjunct MC, with greater anti-inflammatory properties in high-dosage group.
[138]	DB, RCT. Adjunct MC (200 mg/day) with RIS	16 weeks	SCZ patients (*n* = 55)	significant decreases in the SANS total score, the PANSS total score, and the PANSS negative symptoms score at week 16 compared to the placebo group. In addition, the minocycline group had a significant decrease in plasma levels of nitric oxide metabolites but no significant difference in changes in plasma levels of IL-1β or TNF-α compared to the placebo group at week 16.	The nitric oxide pathway may correlate with negative symptom improvements.
[170]	DB, CT. Adjunct MC (100 mg twice daily) with CLZ	10 weeks	SCZ and Schizoaffective (*n* = 52)	BPRS psychosis factor, total score NS. Global cognitive function NS. Working memory improvement, avolition and the BPRS anxiety/depression factor are significant (*p* < 0.05). Fewer headaches and less constipation.	Cognitive subdomains, side effects, negative symptoms, but not overall.
[171]	DB, RCT. Adjunct MC (200 mg/day) with RIS	16 weeks	SCZ patients (*n* = 55)	No improvements on body metabolism, e.g., fasting insulin, lipids, glucose, BMI, waist circumference, and body weight.	
**(b)**					
**Study**				**Outcome**	
Symptom Domains					
[138,152,154]				Total score improvement	
[152,154]				+ve symptom improvement	
[138,152,153,154,162,163,164]				−ve symptom improvement	
[170]: Avolition				Improvements in some domains of −ve symptom	
[163,166]				Cognitive symptom improvement	
[164,170]				Improvement in some domains of the cognitive domain	
Inflammatory Markers					
[166]				Significant reduction in pro-inflammatory cytokines	
[165,171]				No differences in pro-inflammatory cytokines	

BPRS, Brief Psychiatric Rating Scale; CGI, Clinical Global Impression; CLZ, Clozapine; CT, Clinical Trial; DB, Double-blinded; IL, Interleukin; MC, Minocycline; PANSS, Positive and Negative Syndrome Scale; RCT, Randomized Clinical Trial; RIS, Risperidone; SANS, Scale for the Assessment of Negative Symptoms; SCZ, Schizophrenia; TNF-α, Tumor Necrosis Factor α. Improvements compared to baseline, significantly more improvement than the control groups who did not take Minocycline. +ve, positive; −ve, negative.

### 8.4. Exercise and Schizophrenia

Exercise has been widely recognized as a non-pharmacological intervention that promotes neurogenesis and synaptic plasticity. Aerobic exercise, particularly at moderate to high intensity, enhances neuronal survival and connectivity [149].

Clinical and preclinical studies have demonstrated exercise-induced improvements across multiple symptom domains. High-risk individuals for schizophrenia exhibited enhanced episodic memory and recognition accuracy after 3 months of intervention [149]. Meta-analyses have confirmed significant amelioration of positive, negative, or general symptoms of SCZ following sustained exercise programs [150,172,173]. Exercise also improves spatial learning [174] and reverses memory deficits in mice [175] and strengthens hippocampus-dependent functions such as working and long-term memory in patients [149,176]. Yoga, in particular, is associated with improvements in long-term memory [172]. Most prominently, exercise interventions can enhance the confidence and social functioning of psychotic patients, facilitating psychosocial recovery [177].

However, a recent Mendelian randomization (MR) analysis challenges the presumed causal protective role of exercise. Using large-scale genetic datasets, the study found no definitive evidence for exercise-mediated reduction in schizophrenia risk; in fact, moderate to vigorous physical activity was associated with a slightly increased risk, possibly reflecting confounding by symptom severity [178]. Despite these findings, the clinical benefits of exercise for symptom management and cognitive enhancement remain robust.

## 9. Limitations

While this review integrates neurogenesis, neuroinflammation, gut microbiota, and hormonal dynamics in schizophrenia, several limitations must be acknowledged. Current findings are largely correlational, with causality remaining unestablished. Elevated cortisol or androgen levels, for example, may reflect a disease state rather than a primary etiology. Advanced statistical tools such as Mendelian randomization can help elucidate causal pathways between biological mechanisms and clinical outcomes.

**Figure 3:**
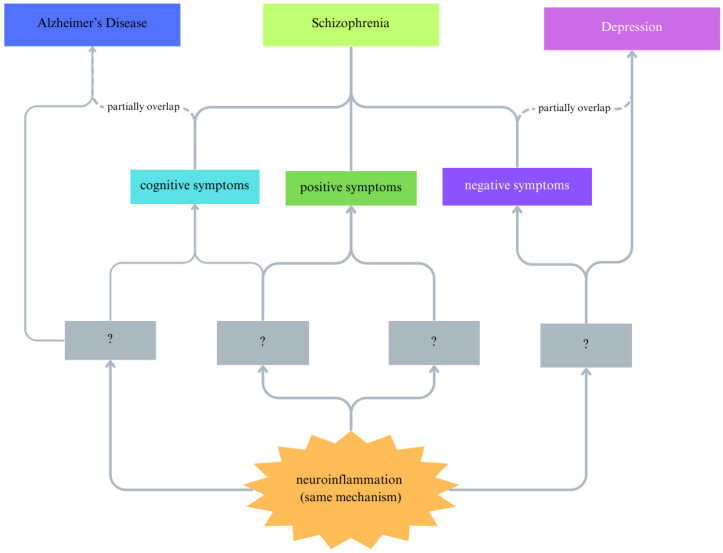
Study Methodology for SCZ and Other Neuropsychiatric Disorders. Shared pathological processes such as neuroinflammation manifest differently across neuropsychiatric disorders (e.g., Alzheimer’s disease, depression, and schizophrenia). Identifying disorder- or symptom-specific molecular signatures will be crucial for discerning causal mechanisms and tailoring therapeutic strategies.

Another limitation lies in the transdiagnostic nature of many implicated pathways. Chronic neuroinflammation, for example, is common across Alzheimer’s disease [179], depression, and prolonged stress exposure, raising questions about specificity to schizophrenia. But how chronic neuroinflammation could impact different pathological statuses and thus lead to various disorders with different presentations is yet to be explored. (Figure 3) Future studies should therefore focus on symptom-specific pathophysiology, identifying biomarkers and mechanisms linked to discrete clinical domains—such as hallucinations or cognitive deficits. Elucidating disease-specific molecular signatures within shared mechanisms will be essential for developing targeted and precision-based therapies.

## 10. Conclusions

The etiology of schizophrenia remains a conundrum, reflecting its multifactorial biological complexity. This paper has synthesized evidence supporting the roles of impaired neurogenesis, dysregulated immune responses, altered gut microbiota, and hormonal disturbances in schizophrenia pathogenesis. Integrating these domains reveals a dynamic interplay among neurodevelopmental, inflammatory, and endocrine mechanisms that collectively shape disease onset and progression. Future therapeutic strategies should therefore extend beyond symptom suppression to address underlying neuropathological processes. By targeting these interconnected systems—through pharmacological, cellular, and lifestyle-based interventions—it may be possible not only to alleviate symptoms but also to promote functional recovery and neurobiological resilience. A mechanistic understanding of these pathways will also inform broader insights into related neuropsychiatric disorders and advance the pursuit of curative treatments for schizophrenia.

## Figures and Tables

**Table 1:** An overview of the systematic reviews on probiotic treatment in SCZ.

Study	Conclusion	Studies Included
[47]	No significant differences post-intervention in overall, positive, or negative symptoms	3
[60]	Reduction in PANSS total score (SMD: −0.608, *p* = 0.035)	5
[61]	Reduction in CRP levels (SMD: −0.46, *p* = 0.001)	4
[62]	Reduction in total, general, and negative scores	6

CRP: C-reactive Protein; PANSS: Positive and Negative Syndrome Scale (PANSS); SMD: Standardized Mean Difference.

**Table 2:** Summary of the future interventions to alleviate schizophrenia and correlated pathways.

		Phase 2: Primary Pathology		Phase 3: Secondary Pathology		Phase 4: Phenotype
Interventions	Primary Target	OS and Neuroinflam	HPA Axis	Neurogenesis	Neurochemistry	Symptomatic Improvements
Atypical Antipsychotics	Neurochemistry	↓ oxidative products [122]; ↓ nitric oxide, IL-1β, IL-6, and TNF-α [123]	↓ basal cortisol [65]	↑ survival [124]; ↑ BDNF expression [125]; ↑ newborn neurons [126]; ↑ 17β-estradiol [127]	D2R and 5-HT 2AR antagonist [128]	−ve symptoms [128]
Exogenous NSCs	Neurogenesis [129,130,131,132,133]	↓ symptomatic inflammation [133]	/	↑ Akt; ↑ survival [124]; ↑ proliferation [134]	/	spatial working memory [135]
Exogenous MSCs		↓ IL-1β; ↑ IL-17 [133]		↑ hippocampal neurogenesis [125]	/	social and cognitive symptoms [125]
Minocycline, adjunct (NSAID)	Neuroinflam	↓ IL-1β, IL-6 and TNF-α, nitric oxide metabolites (mixed results) [124,125,126,127,136,137]; ↑ M2 microglia [138,139]	/	↑ neurogenesis; ↑ BDNF [138,139]	/	−ve symptoms, depressive behaviours [124,125,126,127,136,137]
Vitamin D	Neuroinflam	↓ CRP [140]; ↓ lipid peroxidation [141]; ↑ TAS [141]	/	/	/	Cognitive symptoms [140], +ve symptoms [142]
Exercise	Overall neuroprotective	↓ free radicals [143]; + TH2 profile [143]; ↑ M2 microglia [144]	/	↑ proliferation [145]; ↑ survival [145]; ↑ BDNF [146]; ↑ dendritic density and morphology [147]; ↑ connectivity; ↑ maturation of newborn neurons [146]	↑ glutamatergic [148]	Episodic memory [149], recognition accuracy [149], spatial learning [150], working memory [149], long-term memory [151]; +ve symptoms; −ve symptoms; general symptoms [140,152,153,154]

Akt, Protein Kinase B; BDNF, Brain-Derived Neurotrophic Factor; CRP, C-Reactive Protein; HPA, Hypothalamus–Pituitary–Adrenal; IL, Interleukin; MSC, Mesenchymal Stem Cell; Neuroinflam, Neuroinflammation; NSAID, Non-Steroid Anti-Inflammatory Drug; NSC, Neural Stem Cell; TAS, Total Antioxidant Capacity; TNF-α, Tumor Necrosis Factor α; +ve, Positive; −ve, Negative; +, Activates; ↑, Increases; ↓, Decreases.

## Data Availability

No new data were created or analyzed in this study. Data sharing is not applicable to this article.

## Abbreviations

5-HT	5-Hydroxytryptamine
ACTH	Adrenocorticosteroid Hormone
AD	Alzheimer’s Disease
AD/HD	Attention-deficit Hyperactivity Disorder
Akt	Protein kinase A
APP	Atypical Antipsychotics
AR	Androgen receptor
BBB	Blood–brain barrier
BDNF	Brain-derived neurotrophic factor
BPRS	Brief Psychiatric Rating Scale
CAR	Cortisol awakening response
CNS	Central nervous system
CRH	Corticosteroid-releasing hormone
DA	Dopamine
DHEA	Dehydroepiandrosterone
DISC1	Disrupted in Schizophrenia 1
DKK1	Dickkopf 1
EPS	Extrapyramidal symptoms
ERK	Extracellular signal-regulated kinase
FEP	First-episode psychosis
hiPSC	Human-induced pluripotent stem cell
HPA	Hypothalamus–pituitary–adrenal
GABA	Γ-aminobutyric acid
GPER	G-protein estrogen receptor
GSK-3β	Glycogen-synthetic kinase 3β
IFN-γ	Interferon gamma
IGF-1	Insulin growth factor-1
IL	Interleukin
LTP	Long-term potentiation
MAPK	Mitogen-activated protein kinase
MD	Mitochondrial dysfunction
MDD	Major Depressive Disorder
MR	Mendelian Randomization
MSC	Mesenchymal stem cell
NF-κB	Necrosis factor kappa B
NMDAR	N-methyl-D-aspartate glutamatergic receptor
NSAID	Non-steroid anti-inflammatory drug
NSC	Neural stem cell
OS	Oxidative stress
PDGF	Platelet-derived growth factor
PKA	Protein kinase A
PI3K	Phosphoinositide 3-kinases
ROS	Reactive oxidative species
RNS	Reactive nitrogen species
SCZ	Schizophrenia
TAS	Total antioxidant capacity
TGF	Transforming Growth Factor
TH2	T helper 2 cells
TNF-α	Tumor necrosis factor alpha
TrkB	Tropomyosin receptor kinase B
Wnt	Wingless-type MMTV integration site family
